# Clinical Pharmacokinetic Evaluation of Optimized Liquisolid Tablets as a Potential Therapy for Male Sexual Dysfunction

**DOI:** 10.3390/pharmaceutics12121187

**Published:** 2020-12-07

**Authors:** Fayez O. Alotaibi, Nabil A. Alhakamy, Abdelsattar M. Omar, Khalid M. El-Say

**Affiliations:** 1 Department of Pharmaceutics, Faculty of Pharmacy, King Abdulaziz University, Jeddah 21589, Saudi Arabia; ac-jeddah@hotmail.co.uk (F.O.A.); nalhakamy@kau.edu.sa (N.A.A.); 2 Center of Excellence for Drug Research and Pharmaceutical Industries, King Abdulaziz University, Jeddah 21589, Saudi Arabia; 3 Department of Pharmaceutical Chemistry, Faculty of Pharmacy, King Abdulaziz University, Jeddah 21589, Saudi Arabia; asmansour@kau.edu.sa; 4 Department of Pharmaceutical Chemistry, Faculty of Pharmacy, Al-Azhar University, Cairo 11884, Egypt

**Keywords:** Box-Behnken design, dapoxetine, liquisolid tablet, in vitro dissolution, male sexual dysfunction, tadalafil, pharmacokinetics

## Abstract

The study aimed at developing a liquisolid tablet (LST) containing tadalafil (TDL) and dapoxetine (DPX) with improved bioavailability as a potential therapy for male sexual dysfunction. A mixture of nonvolatile solvents, namely PEG 200 and Labrasol®, was utilized to prepare LSTs that were assessed for their quality characteristics. The Box–Behnken design (BBD) was employed to statistically explore the effect of the formulation factors on the quality attributes of LSTs. Furthermore, an in vivo pharmacokinetic study was carried out for the optimized LST in comparison with the marketed tablets on healthy human volunteers. The optimized LST revealed acceptable quality limits with enhanced dissolution for both APIs. The pharmacokinetic parameters after oral administration of the optimized LST indicated that the Cmax of TDL in LSTs was 122.61 ng/mL within 2h compared to the marketed tablets, which reached 91.72 ng/mL after 3 h, indicating the faster onset of action. The AUC was improved for TDL in LST (4484.953 vs. 2994.611 ng/mL∙h in the marketed tablet) and DPX in LST (919.633 vs. 794.699 ng/mL∙h in the marketed tablet). This enhancement in bioavailability potentially minimizes the associated side effects and improves the treatment of male sexual dysfunction, particularly for diabetic patients.

## 1. Introduction

Male sexual dysfunction (MSD) is a mixed group of complaints that are typically related to a person’s inability to respond sexually or to achieve sexual stimulation. Male sexual dysfunction is a common problem mainly associated with erectile dysfunction (ED) and/or premature ejaculation (PE). It was reported that by 2025 ED is expected to affect about 322 million men around the world. ED is not life-threatening, but it can affect the quality of life of sexual partners and lead to some emotional problems such as anxiety and depression. ED clearly can lead to isolation and prevention between the couple and sometimes these problems can be extended to their jobs and cause negative interaction with others [1]. In addition, the prevalence of PE ranged from 19% to 30% in the general population [2,3]. Regardless of the type of PE, it is usually associated with psychological difficulties that may happen to a person and this will reduce his sexual desire. It affects the quality of a partner’s life that affects his sexual satisfaction, confidence, and interpersonal relationships [4,5,6]. About one-hundred million men worldwide cannot reach satisfactory sexual performance related to the failure of an adequate erection. Patient numbers with male sexual dysfunction will increase to double their current amount in the next twenty-five years [7]. Therefore, it is very important to treat a person that suffers from sexual dysfunction because this issue will lead to a deteriorating partner’s life quality [8].

It is worth mentioning that PE is present in up to 30% of men with ED [9]. ED and PE are not separate things but should be considered as an interconnected case of sexual dysfunction. This point of view supports sexual health care professionals in providing the most suitable therapeutic tactic to improve patient-related results in sexual medicine [10]. It was reported that ED was strongly associated with PE among men with diabetes [11]. PE and ED often coexist and are connected with psychosocial anguish, which tourist attractions the vital role of screening for their co-existence and the need for combined therapy [12].

A potent and selective phosphodiesterase-5 inhibitor (PDE5-I), tadalafil (TDL) is one of the most efficient medicines for the treatment of ED [13]. On the other hand, a selective serotonin reuptake inhibitor (SSRI), dapoxetine (DPX) is newly approved for the treatment of PE [14,15]. It is the first and unique oral pharmacological agent used, and the only SSRI approved in more than 60 countries to treat men with PE [16]. On comparing the on-demand dosing of DPX alone and combined with PDE5-I in subjects with PE and without ED, it was found that a low dose of DPX combined with PDE5-I showed better outcomes compared with that of DPX only. This finding supports the recommendation that the PDE5-Is have a potential role in the treatment of PE without ED [17]. Besides, the combined use of SSRIs and PDE5-Is provided additive favorable effects in men with PE compared with SSRIs or PDE5-I monotherapy [18]. Moreover, DPX provided a remarkable treatment benefit in men with PE and comorbid ED on a stable regimen of PDE5-I [19]. Finally, it was reported that the combination of DPX with TDL is well tolerated and the concomitant administration of TDL and DPX did not affect the pharmacokinetics of both APIs [20].

However, TDL undergoes low bioavailability due to its inherent poor aqueous solubility [21,22]. In addition, DPX was suffering from low and variable oral bioavailability that ranges from 15 to 76% [23]. This low and variable drug concentration in the blood may lead to a decrease in their efficacy and/or exaggerated side effects.

To overwhelm this hurdle that encounters the formulators of the oral solid dosage form, many researchers have developed various approaches to improve drug water-solubility. From these approaches, adjustment of the pH, the addition of cosolvent, particle size reduction, solid dispersion [24], salt formation or formulation of the drug in lipid-based nanovesicles such as liposomes [25], nanosuspension development [26,27], prodrug synthesis [28], the formation of micro- and nano-particles [29], or incorporation of drugs into the porous structure and nanoemulsion formulations [30,31]. However, the liquisolid (LS) technique is a promising approach used to enhance the solubility of drugs. When the patient takes drugs orally in a state of a solid dosage form such as capsule or tablet, it should be released and dissolved in gastrointestinal fluids before absorbing to give its desired therapeutic effects. Many poorly water-soluble drugs have a limitation of bioavailability related to their dissolution rate. The technique is used to convert a liquid into easily compressible, non-adherent, and free-flowing dry powder by mixing with selected appropriate excipients known as carrier materials (such as cellulose, starch, lactose, and Avicel PH 101) and coating materials (such as silica powder). It is used to increase drug release properties, hence the bioavailability of water-insoluble drugs due to the observed increase in the surface area of the drug and the wetting properties available for dissolution. The LS technique has contributed to diminishing the effect of pH changes on drug release [23,25,32].

Therefore, the objective of the present study was to develop an optimized combined-dose medication containing TDL and DPX as an effective therapy for male sexual dysfunction. To achieve this aim, the BBD will be used to investigate the effect of different formulation factors on the quality of LSTs that subsequently affect the dissolution and the bioavailability of TDL and DPX.

## 2. Materials and Methods

TDL was gifted from SAJA Pharmaceutical Co. Ltd. (Jeddah, Saudi Arabia). DPX was kindly gifted from Spimaco Addwaeih (Riyadh, Saudi Arabia). Glycerine was supplied by Crescent Diagnostics (Jeddah, Saudi Arabia). Cellulose microcrystalline (Avicel^®^ PH-101), sorbitan monooleate 80 (Span^®^ 80), Macrogolglycerol ricinoleate; Kolliphor^®^ EL (Cremophor^®^ EL), and Polysorbate 80 (Tween^®^ 80) were purchased from Sigma Aldrich (Steinheim, Germany). Silica fumed anhydride, amorphous silicon, and silicon dioxide was obtained from Sigma-Aldrich (St. Louis, MO, USA). Polyethylene glycol (PEG) 200 was purchased from BDH Limited (Poole, England). Polyethylene glycol 400 was purchased from Across Organics (Morris Plains, NJ, USA). Caprylocaproyl macrogol-8/polyoxyl-8 glyceride (Labrasol^®^) was supplied by Gattefosse’ (Saint-Priest Cedex, France). Crospovidone NF (Polyplasdone XL-10) was supplied by ISP Technologies (Ashland, KY, USA). Methanol was purchased from Honeywell (Seelze, Germany). Magnesium stearate was purchased from (Prolabo, France). Magnesium Trisilicate was purchased from Loba Chemie Pvt. Ltd. (Mumbai, India). Talc powder was purchased from Qualigens fine chemicals (Mumbai, India).

### 2.1. Pre-Formulation Studies

#### 2.1.1. Solubility Studies

Solubility studies of TDL and DPX were determined in various nonvolatile solvents separately, as we described previously with full details published [23,33,34]. The solvents used in this study were Span 80, Tween 80, PEG 400, PEG 200, Labrasol^®^, Kolliphor^®^ EL, glycerin, and distilled water. The solubility of both drugs in all solvents was determined three times and the data were expressed as the average ± the standard deviation.

#### 2.1.2. Holding Capacity and Determination of the Liquid Load Factor (L_f_)

The holding capacity of the liquisolid excipients is the maximum amount of a non-volatile liquid that can be held inside the bulk of the carrier and coating powders while maintaining an acceptable flowability that was obtained by using the previously reported method [23]. Briefly, different weights (0.5, 1.0, 1.5, 2.0 and 2.5 g) of solvents; PEG 200 and Labrasol^®^; were added to different mortars containing 5 g of Avicel PH-101 and triturated well. Then 0.5 g that gradually increased to 1.0 g of silica was added and triturated to give good distribution for the liquid through the powder blend. Powder addition and trituration were sustained up until mortar contents began to appear as a dry powder. This procedure was repeated by increasing the weight of silica in the powder to 1.0 g to evaluate if there is an improvement in the flowability of powder blends achieved or not. Finally, the liquid load factor (L_f_) which possesses an acceptable flowable and compressible blend, was determined. The liquid load factor (L_f_) was calculated by dividing the liquid medication weight (W) by the carrier powder weight (Q) in the system (i.e., L_f_ = W/Q). The flowability of the prepared mixtures was examined according to the U.S. Pharmacopeia as described in the General Chapters: <1174> POWDER FLOW either by direct technique through the determination of the angle of repose, or with the indirect method by calculation of both Hausner ratio and Carr’s index after measuring the bulk and tap densities of the LS powder blends as described in Equations (1)–(3).

#### 2.1.3. Solid-State Characterization Studies

##### Differential Scanning Calorimetry (DSC)

DSC was carried out to evaluate the thermotropic characteristics and thermal performance of TDL and DPX and the LS compacts using a DSC 8000, PerkinElmer, Inc. (Waltham, MA, USA). About 5 mg of the sample was sealed in aluminum pans and heated at the rate of 10 °C/min, covering a temperature range of 25–400 °C under a nitrogen atmosphere at a flow rate of 100 mL/min.

#### Fourier Transform Infrared Spectroscopy (FT-IR)

To investigate potential interactions between TDL and DPX with the tablet’s excipients in the LST, FT-IR spectra were obtained using a Nicolet iS10, Thermo Scientific Inc., (Waltham, MA, USA).

#### Powder X-ray Diffraction (PXRD)

The crystallinity of LS powder formulations was considered using PXRD. PXRD diffractograms for TDL and DPX and the prepared LS system were determined using the Ultima IV diffractometer (Rigaku Inc., Tokyo, Japan).

### 2.2. Formulation of TDL and DPX Liquisolid Tablets

BBD was employed to evaluate the effect of the liquid load factor (L_f_) as X_1_, the powder excipient ratio (R) as X_2_, and the superdisintegrant (Polyplasone XL-10) percentage as X_3_ on the quality attributes of the LS formulations. Fifteen experimental runs were suggested by the design. X_1_ was studied in the level from 0.2 to 0.4, X_2_ from 5 to 15, while X_3_ was studied from 4 to 6%. Statistical analysis was performed using Statgraphics Centurion 18 software, Statgraphics Technologies, Inc. (Virginia, VA, USA) to investigate the effect of these independent variables on the tablet hardness (Y_1_), the disintegration time (Y_2_), the dissolution efficiency percent of TDL after 60 min (Y_3_) and the dissolution efficiency percent of DPX after 60 min (Y_4_).

### 2.3. Preparation of TDL and DPX Liquisolid Tablets

Fifteen formulations of LSTs (LS-1 to LS-15) were prepared as shown in Table 1. Briefly, 100 mg of TDL was dissolved in the first half of the calculated solvent mixture (PEG 200) and 600 mg of DPX was dissolved in the second half of the solvent mixture (Labrasol^®^) and the mixture was mixed well. The calculated amounts of Avicel PH 101, fumed silica, magnesium trisilicate, polyplasdone XL-10, and Methocel^®^ ES were added to the mixture with continuous trituration for 10 min in a mortar and the dried mixture were passed through 20 mesh sieves. Finally, the mixture was mixed with 0.5% of talc and magnesium stearate. The obtained powder blend of the fifteen formulations was examined for the flowability parameters before their compression into LSTs. The powder excipients were de-lumped individually through a No. 40 mesh sieve. The de-lumped powders were mixed for 15 min. Talc powder and magnesium stearate were also de-lumped through the 40-mesh sieve and then added to the powder blend and mixed for 3 min. The LSTs were made at 10 KN compression force in a single punch tablet press (Erweka, GmbH, Heusenstamm, Germany) equipped with 9 mm flat round tooling sets.

### 2.4. Pre-Compression Evaluation of the Liquisolid Powder Formulations

Each LS powder blend was evaluated physically before compression into a tablet by determination of the angle of repose (direct method), bulk and tap density, calculation of Hausner ratio, and Car’s index (compressibility percent) from the Equations (1)–(3) [35,36]. The angle of repose was determined according to Equation (1). Where H is the height and D is the mean diameter of the cone.

(1) $$\mathrm{Tan}\;\left(\theta \right)=(\frac{2\;H}{D})$$

The Hausner ratio and Carr’s index were calculated from Equations (2) and (3).

(2) $$\mathrm{Hausner}\;\mathrm{Ratio}=\frac{\mathrm{Tapped}\;\mathrm{Density}}{\mathrm{Bulk}\;\mathrm{Density}}$$

(3) $${\mathrm{Carr}}^{\prime }s\;\mathrm{Index}=(\frac{\mathrm{Tapped}\;\mathrm{Density}-\mathrm{Bulk}\;\mathrm{Density}}{\mathrm{Tapped}\;\mathrm{Density}})\;\times 100$$

### 2.5. Post-Compression Evaluation of the Prepared Liquisolid Tablets

The LS tablets were visually inspected for any drawbacks during the compression and then examined for their quality attributes, such as weight and content uniformity, thickness, hardness, friability, and disintegration time, according to the requirements of the United States Pharmacopeia [37].

#### 2.5.1. In-Vitro Disintegration Study

The study was carried out on 6 tablets using a Pharma test disintegration tester according to the USP specifications. Distilled water at 37 ± 0.5 °C was used as a disintegration media and the time in seconds taken for complete disintegration of six tablets was recorded and the average of 6 determinations was reported.

#### 2.5.2. In-Vitro Dissolution Study

The study was performed with the dissolution apparatus II (paddle type). The dissolution medium of 900 mL distilled water at 37 ± 0.5 °C at a rotation of 100 rpm was used. Aliquots of 5 mL were withdrawn at predetermined time intervals 5, 10, 15, 20, 30, 45 and 60 min and filtered through a 0.45 µm Millipore filter (Millipore Corp., Bedford, MA, USA). The concentrations of TDL and DPX were determined spectrophotometrically at 284 and 291 nm, respectively, using a UV–Vis spectrophotometer (Jenway 7315, Bibby scientific Limited, Stone, Staffordshire, UK).

#### 2.5.3. Mathematical Modeling of the Dissolution Data

The in vitro dissolution of TDL and DPX from the fifteen LST formulations and the optimized LSTs formulation were fitted to Zero [38], First [39], Weibull [40], Hixson–Crowell [41], Higuchi [42], Korsmeyer–Peppas [43] and Baker–Lonsdale [44]. The highest value of the coefficient R^2^ was used to identify the goodness of fit and the appropriate release model.

#### Dissolution Rate (DR_10_)

For comparative evaluation, TDL and DPX dissolution rates (D_R10_) for the LS formulations were used [45,46]. The amount of TDL and DPX (in µg) dissolved per min during the first 10 min, was calculated from Equation (4).

(4) $${\mathrm{DR}}_{10}=\frac{M\times D}{1000}$$

where M is the total amount of TDL and DPX in each tablet (in this study it is 2500 and 15,000 µg for TDL and DPX, respectively) and D designates the percentage of drug dissolved during the first 10 min. In addition, other non-model parameters such as the mean dissolution time (MDT) and the dissolution efficiency at 60 min (DE_60_) were calculated from the in vitro release data using DDSolver (an add-in program for modeling and comparison of drug dissolution profiles) [47].

#### Mean Dissolution Time (MDT)

MDT is defined as the mean residence time of a drug in the formulation [48]. It is a beneficial parameter for describing the drug release rate from a dosage form and is calculated using Equation (5).

(5) $$\mathrm{MDT}=\frac{\sum_{j=1}^{n}t_{j}^{\mathrm{AV}}\times \Delta Q_{j}}{\sum_{j=1}^{n}\Delta Q_{j}}$$

where (j) is the sample number, *n* is the number of dissolution sample times, ($t_{j}^{\mathrm{AV}}$) is the time at the midpoint between t and t − 1 (calculated with (t + t − 1)/2) and ($\Delta Q_{j}$) is the additional amount of drug dissolved between t and t − 1.

#### Dissolution Efficiency after 60 Min (DE_60_)

DE_60_% expresses the integrated area under the dissolution curve up to a certain time, t, as a percentage of rectangle area represents 100% dissolution at the same time [49,50]. The DE of the formulations was calculated using Equation (6).

(6) $$\mathrm{DE}=\frac{\int_{0}^{t}\mathrm{Qdt}}{Q_{100}\times t}\times 100$$

where (Q) is the percent of drug released as a function of time, (t) is the total time of drug release, and (Q_100_) is 100% drug release.

### 2.6. Prediction of the Optimized Formulation

Analysis of variance and multiple response optimization developed for responses showed the effect of each input variable and their interactions with other variables were utilized for predicting and obtaining the optimized TDL and DPX LSTs using the statistical package Statgraphics^®^ Centurion 18 Software (StatPoint, Inc., Warrenton, VA, USA). The optimized formulation was prepared and fully characterized by the hardness, disintegration time, and dissolution efficiency after 60 min. This optimized formulation was scaled up to be evaluated in vivo for its pharmacokinetic parameters on human volunteers.

### 2.7. In-Vivo Pharmacokinetic Evaluation on Healthy Human Volunteers

A pharmacokinetic study was carried out for the optimized LST (test) in comparison with the marketed tablet (reference) on healthy human volunteers. The prepared LSTs and the marketed tablets were administered orally.

#### 2.7.1. Study Design and Conduct

A single-dose one-period parallel design was used in the study. The study was performed following EMA (European Medicines Agency), ICH (International Conference on Harmonization), GCP (Good Clinical Practice), and FDA (Food and Drug Administration) guidelines. The Protocol was approved by the Egyptian Research and Development Company Research Ethics Committee (ERDC REC), Cairo, Egypt, on its expedited meeting on the 2nd of July 2018 with the Ethical Approval Code (0569/452). Six subjects per group gave written informed consent before participation in this study. The subjects who participated were in good health as determined by past medical history, physical examination, vital signs and laboratory tests (hematology, biochemistry, and urine analysis). They were also screened for viral infections and remained under close medical supervision until 24 h after the study period. Each subject fasted for at least 12 h before the administration of tablets. Subjects were kept in-house for 72 h before and after administration of the drug so that regular blood sampling could be withdrawn at a predetermined time (as described in the “Blood Sampling” section).

#### 2.7.2. Subjects

Twelve healthy Egyptian male volunteers participated in the study. The age and body mass index (BMI) of the subjects ranged from 21 to 30 years and 20 to 30 kg/m^2^, respectively, with a median height of 172 ± 5.3 cm. Subjects were classified into two groups (6 per group); the first group was administered the optimized TDL and DPX LST, and the second group was given the marketed film-coated tablets. The marketed tablets used were TDL 5 mg (Cialis 5 mg, Lilly S.A., Madrid, Spain) and DPX 30 mg (Joypox 30 mg tablets that were produced by South Egypt Drug Industries Co. (SEDICO, Cairo, Egypt).

#### 2.7.3. Blood Sampling

A sample of 5 mL of blood was drawn just before and at 0.25, 0.5, 0.75, 1, 1.5, 2, 2.5, 3, 4, 6, 8, 10, 12, 24, 36, 48, 60 and 72 h after the oral administration of both the test and reference and collected in heparinized tubes. Samples were centrifuged at 3000 rpm for 5 min and plasma samples were collected and stored at −20 °C until analysis.

#### 2.7.4. Chromatographic Conditions

A high-performance liquid chromatographic method coupled with MS/MS detection (HPLC-MS/MS) was developed at ERDC laboratories for the determination of TDL and DPX in human plasma. Agilent series 1200, Agilent Technologies, Deutschland GmbH, (Waldbronn, Germany), equipped with G1311A quaternary pump, G1329A, autosampler, G1322A vacuum degasser, and mass hunter software were used. Chromatography was performed using 75% acetonitrile to 25% of 10 mmoles of ammonium acetate and 100 uL formic acid for each 100 mL water as the mobile phase at a flow rate of 0.3 mL/min and the reverse phase column Intersil ODS-3 (4.6 mm × 50 cm, dp 5µm) from (GL Sciences, Tokyo, Japan) temporized at 25 °C. Sildenafil was used as an internal standard (IS). TDL, DPX, and Sildenafil (IS) were monitored by applying positive multiple reaction monitoring (+MRM). The protonated precursor ions and the produced ions were m/z 390.1(268.0), m/z 306.1 (261.0), and m/z 475.3 (100.1), respectively. The retention time was 2.25, 2.32 and 2.17 min for TDL, DPX, and Sildenafil (IS), respectively. The linearity of the assay for TDL was verified within the concentration range of 1–200 ng/mL with a regression coefficient R^2^ = 0.998. All the results were within the acceptance criteria as stated in the recommended guidelines. The mean recovery of TDL was 100% at 1 ng/mL (lower limit of quantification; LLOQ) and 95.3% at 200 ng/mL (upper limit of quantification; ULOQ). The described method is proved to be sensitive, accurate, and reproducible with a lower limit of quantification of 1 ng/mL for TDL, while the linearity of the assay for DPX was verified within the concentration range of 2–500 ng/mL with a regression coefficient R^2^ = 0.994. All the results were within the acceptance criteria as stated in the recommended guidelines. The mean recovery of DPX was 99.8% at 2 ng/mL (LLOQ) and 92.2% at 500 ng/mL (ULOQ). The described method is proven to be sensitive, accurate, and reproducible with a lower limit of quantification of 5 ng/mL for DPX.

#### 2.7.5. Pharmacokinetic Data Analysis

The plasma concentration of TDL and DPX versus time and the pharmacokinetic parameters were determined by the non-compartmental pharmacokinetic model using PKsolver (An add-in program for pharmacokinetic data). Maximum (peak) plasma concentration over the time specified (C_max_), and time point of maximum plasma concentration (T_max_), and the area under the plasma concentration–time curve from zero time to the last measurable concentration (AUC_0–t_) was calculated by the linear trapezoidal method. In addition, individual estimates were made of the terminal elimination rate constant (Lambda_z), the mean residence time (MRT_0-inf_) which was calculated by the ratio of AUMC to AUC, and the elimination half-life (t_1/2_) which was calculated as 0.693/Lambda_z. Moreover, the apparent total body clearance of the drug from plasma after oral administration (Cl/F) was calculated by dividing the dose by AUC and the apparent volume of distribution during the terminal phase after non-intravenous administration (Vz/F) was calculated by multiplying total body clearance by MRT. Finally, the relative bioavailability of the optimized LSTs (AUC test/AUC standard ×100) was determined.

### 2.8. Statistical Analysis

All statistical analyses were performed using GraphPad Prism 8 for Windows, Version 8.2.1 Software (San Diego, CA, USA). Regarding the plasma concentration–time curve, two-way ANOVA followed by Sidak’s multiple comparisons test was conducted to compare each mean with the others at all time points and assess the significance between groups, while a two-tailed unpaired *t*-test was used to assess the pharmacokinetic parameters of the formulations. Results with *p* < 0.05 were considered significant.

## 3. Results and Discussion

### 3.1. Pre-Formulation Studies

#### 3.1.1. Solubility Study

**Figure 1:**
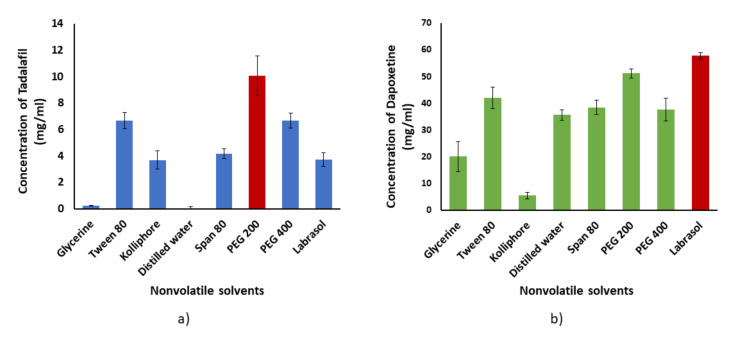
Solubility of (**a**) TDL and (**b**) DPX in different non-volatile solvents. The error bars indicate the standard deviation of three determinations.

Figure 1a illustrates the solubility of TDL in different nonvolatile solvents. PEG 200 showed the highest solubilization capacity of TDL (10.07 mg/mL), while the solubility of TDL in distilled water was 0.049 mg/mL, which confirms that TDL is practically insoluble in water according to USP, which describes the substance that needs more than 10,000 mL to dissolve 1 g with the practically insoluble one.

Figure 1b illustrates the solubility of DPX in different nonvolatile solvents. Labrasol^®^ showed the highest solubilization capacity of DPX (57.71 mg/mL) when compared with other non-volatile solvents in the study. Despite DPX is soluble in water with a solubility value of 35.63 mg/mL; its solubility has been improved markedly with Labrasol, which is favorable in LS formulation.

#### 3.1.2. Holding Capacity and Determination of Liquid Load Factor (L_f_)

The flowability parameters of Avicel PH 101 were assessed after the addition of 0.5 g of silica and after the addition of 1 g of silica at different liquid load factor using different weights of the solvent mixture of PEG 200 and Labrasol^®^ (1:1 *w*/*w*). The flowability parameters of these blends were evaluated to choose the liquid load factor suitable to attain acceptable flowability with maximum drug loading in TDL and DPX LS formulations. All the trials showed values of angle of repose more than 43° which indicates the poor flowability of these powder blends. By increasing the amount of the added silica to 1 g, the angle of repose decreased to be 37°, which confirms the improvement of the flowability of the powder blends. The same finding has been displayed with the results of the Hausner ratio and Carr’s index of the same powder blends as the Hausner ratio values ranged from 1.18 to 1.25 and the Carr’s index from 15 to 20, respectively, which revealed that the addition of 1 g silica to the powder blends was of great value in the improvement of the flowability. This finding could be explained by the addition of nanometer-sized silica to reduce the van der Waals interparticle attractive force between the powder particles which subsequently improves their bulk flow behavior [51]. In addition, this result can be explained by the ability of silica powder to spread on the surface of the other excipient and prevent them from contacting directly [52]. Moreover, a nanometer-sized flow regulating particles such as silica wedged in between two micrometer-sized host particles formed an equilateral triangle lattice structure and reduced the van der Waals attractive force between the host particles dramatically [53].

#### 3.1.3. Solid-State Characterization Studies

##### Differential Scanning Calorimetry (DSC)

**Figure 2:**
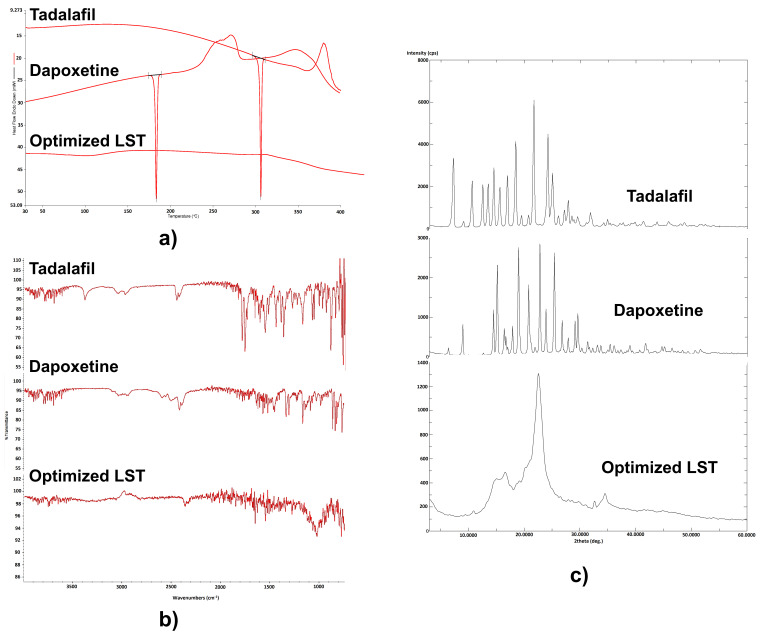
DSC Thermograms (**a**), FTIR Spectra (**b**), and PXRD diffractograms (**c**) of tadalafil, dapoxetine, and optimized liquisolid tablet.

Figure 2a shows the DSC thermograms of raw TDL, raw DPX, and the optimized LS formulation. The obtained thermograms show an endothermic peak around their melting point. In the thermogram of TDL, a sharp endothermic peak corresponding to the melting point of TDL appears at 306.10 °C, which indicates the crystalline nature of TDL, while the thermogram of DPX shows a sharp endothermic peak corresponding to its melting point at 184.02 °C, indicating the crystalline nature of DPX. However, no peak was obtained in the DSC thermogram of the optimized LST, suggesting that the drugs are in a completely solubilized state in the LS system. This observation could be due to the formation of a solid solution in the LS powder form, which means that the drugs are dispersed in the LS matrix at the molecular level [54]. The absence of the endothermic peak can also be attributed to the suppression of the thermal feature of the drug because of the formation of an amorphous solid solution [32].

#### Fourier Transform Infrared Spectroscopy (FTIR)

Figure 2b shows the FTIR spectra of raw TDL, raw DPX, and the LS formulation. Absorption peaks properties for TDL were recorded in the 1800−525 cm^−1^ range. This spectral range contains 1720 to 1150 cm^−1^ domains, important for the finding of TDL analog. The specific TDL absorption bands of the FTIR spectrum were recorded at 1675 cm^−1^ (properties of amides C=O 1646 cm^−1^ (C=C aromatic). The band of 1435 cm^−1^ relates to the stretching vibration C–N, and the band 746 cm^−1^ is representative of benzene [55]. In addition, it can be recognized from Figure 2b, which shows the characteristic DPX peaks of 4000, 3053, 2400, 1500 and 1100. The IR spectrum of Labrasol showed broadband at 2850 cm^−1^, indicating the presence of a hydroxyl group. Additionally, the presence of a carbonyl group was confirmed by the appearance of a characteristic band at 1100 cm^−1^. The hydrogen bonding could shift both hydroxyl and carbonyl groups. The absorption bands of the optimized LS formulation did not show interference with the characteristic drug peaks, indicating the absence of chemical interaction between TDL or DPX with LS formulation excipients and entrapment of drugs inside the carrier matrix of the formulation [56,57].

#### Powder X-ray Diffraction (PXRD)

PXRD is used to determine the crystalline state of drugs in a pure state and LS formulation. It measures the disappearance of constructive specific peaks of drugs in the LS formulation and retaining peaks of the carrier material. The PXRD pattern in Figure 2c of the pure drug (TDL) shows sharp diffraction peaks at 7, 10.5, 11.5, 12.45 and 22° with high intensity, which indicates that the pure drug was present in the crystalline state. The PXRD in Figure 2c demonstrates that pure DPX was clearly in the crystalline state as it shows sharp distinct peaks at 2θ diffraction angles of 9, 15, 18, 21, 23 and 25.5° with high intensity, which indicates that the pure drug is in the crystalline state. The decrease in the number and intensity of characteristic peaks in the XRD pattern of the LS formulation as illustrated in Figure 2c indicates the conversion of both drugs from crystalline to amorphous form. This lack of crystallinity in the formulation might be due to the solubilization of drugs in non-volatile liquid vehicles and subsequent adsorption on Avicel and fumed silica. The solubilization or amorphization of the drug in the LS technique leads to the resulting improvement in the apparent solubility and the dissolution rate of the drug.

### 3.2. Formulation and Evaluation of the Liquisolid Tablets

Fifteen formulations of the LS powder blends were prepared as suggested by BBD (Table 1). All formulations were evaluated before compression for their flow and packing properties and after compression for their quality attributes of the prepared LSTs as discussed in the following sections.

#### 3.2.1. Pre-Compression Evaluation

The micromeritic properties of the pre-compressed LS powder blends, such as Hausner’s ratio, Carr’s index, and the angle of repose, were found to be in the range of 1.09–1.31, 5.1–25 and 21–41°, respectively, which indicate that, the flow properties of the powder blends were acceptable for all formulations. It is obvious from the data in Table 2 that two formulations (LS-11 and LS-15) out of 15 LS formulations had passable flow property because they have the highest values of Hausner ratio, Carr’s index, and angle of repose (1.31, 25 and 41° for LS-11 and LS-15), whereas LS-8, LS-9, LS-12 formulations had fair flow. On the other hand, the rest of the remaining formulations exhibited good to excellent flow behavior as the value of the Hausner ratio was less than 1.2 [35]. In addition, these formulations can be compressed as Carr’s index data showed results of less than 21% [51] and the angle of repose was less than 35.

#### 3.2.2. Post-Compression Evaluation

Quality control tests of the prepared LSTs presented in Table 2 revealed that the TDL content of all formulations was found to be in the range of 95.2% to 103.2% in LS-1 and LS-10, respectively, while the DPX content of all formulations was in the range of 95.6% to 102.2% for LS-10 and LS-13, respectively. These results were compiled with the official specifications of USP and reflect the uniformity of weight in all formulations [58]. In addition, there is no observed variation in the thickness of all formulations. The friability and the hardness of all tablet formulations ranged from 0.086–0.461%, and 32.14–58.02 N, respectively, which complied with BP friability test limits (<1%). The friability and hardness results reflected the acceptable mechanical properties and good breaking strength of the prepared LSTs as well as overcome the poor compressibility of the LS powders. The good results of the tablet hardness may be due to hydrogen bonding between Avicel molecules and PEG 200 [59]. Regarding the in vitro disintegration time for each batch of LSTs, it was found that the mean of the disintegration times for all investigated tablets was less than 10 min, which met the Pharmacopoeial requirements. The disintegration time of the prepared tablets was ranged from 0.26 min for LS-4 to 6.29 min for LS-9, which showed the longest disintegration time.

#### 3.2.3. In-Vitro Dissolution Studies

**Figure 3:**
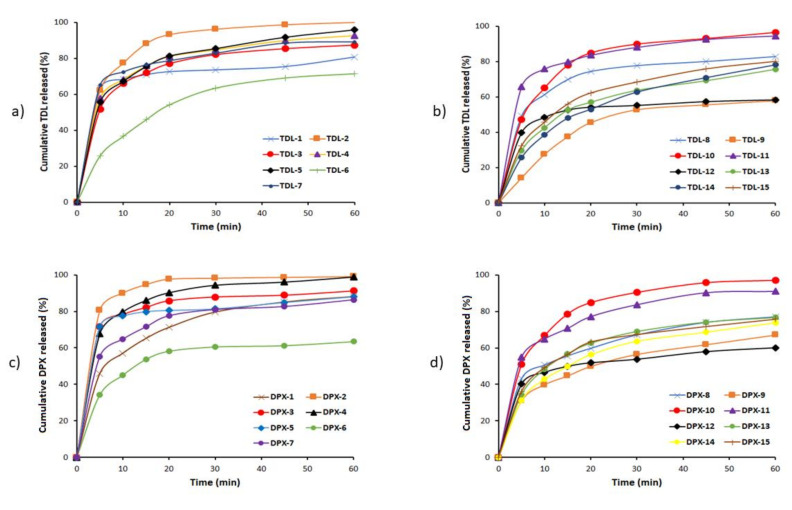
In vitro release profiles of TDL (**a**,**b**) and DPX (**c**,**d**) from the liquisolid formulations.

In-vitro dissolution profiles of the fifteen formulations of LSTs were presented in Figure 3a–d for TDL and DPX, respectively. It was found that, among the fifteen formulations for TDL, LS-2 has a maximum dissolution rate that released 75% of its drug content in the first 10 min and 100% in 60 min followed by LS-11 and LS-7, which released 75.76 and 72.45% during the first 10 min, respectively, and more than 89% after 60 min for both formulations. Most of the prepared formulations released more than 75% of their TDL within 60 min except formula LS-6, LS-9, and LS-12, which released 57.84, 71.45 and 58.34%, respectively, within 60 min. In addition, LS-2 released more than 80% of its DPX content in the first 10 min and 98.8% in 60 min, followed by LS-4 and LS-3, which released 85.97 and 82.20% during the first 10 min and more than 90% during the study period. Most of the prepared LSTs released more than 75% of their DPX within 60 min, except formula LS-6, LS-9, LS-12, and LS-14, which released 63, 67, 60 and 73.8%, respectively.

Results illustrated that there is a relationship between the superdisintegrant and the dissolution profile. Formulations containing a high percentage of superdisintegrant (LS-2) showed the highest cumulative amount of TDL and DPX released. In contrast, the formulations containing a low percentage of superdisintegrant (LS-9 and LS-12) showed the lowest cumulative amount of TDL and DPX released. This result might be attributed to the short disintegration time of LSTs leading to the rapid dissolving of the tablet into small particles, thus increasing the surface area exposed to the medium and enhancing the dissolution and bioavailability of the drug and vice versa [60].

#### 3.2.4. Mathematical Modeling of the Release Data

According to the R^2^ values, the release data of the LS prepared formulations were found to fit the Weibull model that establishes a linear pattern when plotting the logarithm of the amount of drug released against the logarithm of time [61]. In addition, all formulations displayed computed β values greater than 1. Moreover, there is a linear relationship between β values and *n*-values that are used in the Korsmeyer–Peppas model [62].

The dissolution rate after the first 10 min (DR_10_) was taken as a measure of the extent and the rate of drug dissolved from the prepared formulations. The results in the tables affirm that the LS formulations LS-2, LS-11, and LS-7 showed the highest value of DR_10_ with 193.25, 189.42 and 181.15 µg/min, respectively, of its TDL content during the first 10 min, whereas the formulations LS-2, LS-4, and LS-3 showed the highest values of DR_10_ with 1349.79, 1195.44, and 1174.67 µg/min, respectively, of its DPX content during the first 10 min. In addition, the calculated values of both dissolution efficiencies after 60 min (DE_60_%) of TDL and DPX from the LS formulations were reported in Table 3. Moreover, the MDT value of TDL ranged from 6.9 h in LS-12 to 16.57 h in LS-14, whereas the value of MDT was increased from 4.39 h in LS-2 to 13.98 h in LS-9, which could be ordered as a function of the solubility of the drug. DE_60_ values in both drugs increased with an increase in DR_10_ and this indicated that the LS approach markedly enhanced the dissolution rate and efficiency.

### 3.3. Response Surface Methodology for Optimization of the Formulation

RSM has been widely used in the formulation development of modern products and for the modification of existing products. It produces polynomial equations and maps the responses over formulation variables to determine the optimum formulation [63]. This study is based on RSM to recognize the influence of dependent variables (X_1_, X_2_ and X_3_) on different response variables (Y_1_, Y_2_, Y_3_ and Y_4_). Table 3 listed the BBD matrix that involves the independent and dependent variables of all suggested formulations.

#### 3.3.1. Effect of the Independent Variables on the Tablet Hardness (Y_1_)

**Figure 4:**
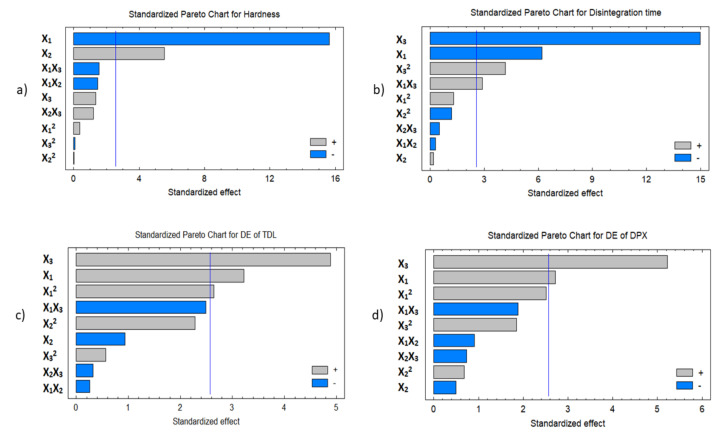
Standardized Pareto charts for the responses (Y_1_–Y_4_, corresponding to **a**–**d**). Abbreviations: X_1_, liquid load factor; X_2_, excipient ratio; X_3_, superdisintegrant concentration; X_1_ X _2_, X_1_X_3_, X_2_ X_3_, the interaction term between the factors; X_1_^2^, X_2_^2^, and X_3_^2^ are the quadratic terms of the factors.

Hardness is a crucial test for evaluating the mechanical durability of LSTs. Table 3 shows the variabilities in the hardness of the prepared LSTs that ranged from 32.14 to 58.01 *n* for LS-11 and LS-1, respectively, on changing the levels of the investigated factors. The estimated effects of the investigated factors and associated *p*-values on the responses are displayed in Table 4 and the standardized Pareto chart in Figure 4. ANOVA results exposed a significant antagonist effect of the liquid load factor (X_1_) on the hardness (Y_1_) with a *p*-value of 0.0001, while the excipient ratio (X_2_) was found to have a significant synergistic effect on Y_1_ with a *p*-value of 0.0026, as presented in Table 4 and Figure 4. The prediction Equation (7) to correlate individual and significant variables with the obtained hardness is shown below:

Hardness (Y_1_) = 45.258 − 22.3 X_1_ + 0.379 X_2_ + 3.366 X_3_ + 32.417 X_1_^2^ − 2.45 X_1_ × 2 − 13.2 X_1_X_3_ − 0.0006 X_2_^2^ + 0.206 X_2_X _3_ − 0.066 X_3_^2^ (7)

**Figure 5:**
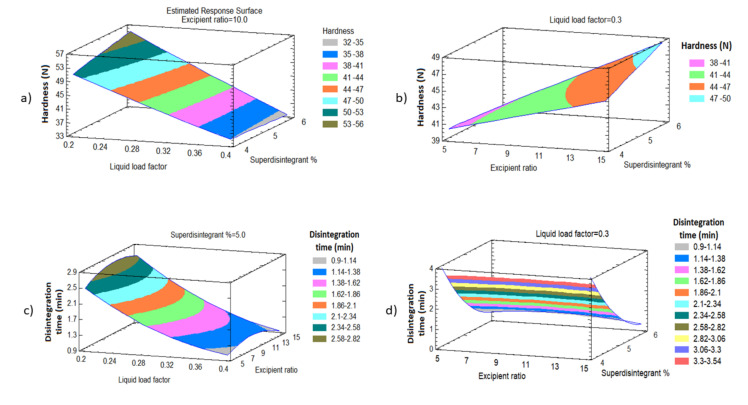
Response surface plots showing the effect of X_1_, X_2_, and X_3_ (**a**,**b**) on the hardness and (**c**,**d**) on the disintegration time.

Figure 5a revealed that there is an inverse relationship between the liquid load factor (X_1_) and the hardness (Y_1_) of the formulations. As the X_1_ increased from 0.2 to 0.4 at the same level as the other factors, the hardness decreased from 58.01 to 36.75 N in LS-1 and LS-7, respectively, and from 56.44 to 35.28 *n* in LS-3 and LS-4, respectively. This trend can be confirmed by the decrease in the hardness from 48.51 to 32.14 in LS-8 and LS-11, respectively, as X_1_ increased from 0.2 to 0.4.

On the other hand, Figure 5b reveals that increasing the excipient ratio percentage (X_2_) in the LSTs showed a significant increase in tablet hardness. The increase in X_2_ from 5 to 15 was always accompanied by an increase in the hardness of tablets. At the same level of both X_1_ and X_3_ and increasing the X_2_ from 5 to 15, the hardness increased from 38.91 to 47.14 N in LS-2 and LS-5, respectively, and from 41.85 to 45.96 N in LS-6 and LS-12, respectively. This observation could be confirmed by the increase in Y_1_ from 48.51 to 58.04 N for LS-8 and LS-1, respectively. This may be attributed to the formation of hydrogen bonding between hydrogen atoms on the adjacent cellulose molecules in Avicel PH-101 that was revealed by DSC, FTIR, and XRD investigation. In addition, the PEG 200 molecule contains more hydroxyl groups, thus there is also a probability of forming hydrogen bonds with Avicel PH-101 [60].

#### 3.3.2. Effect of the Independent Variables on Tablet Disintegration (Y_2_)

Fast disintegration of tablets is necessary to ensure the tablets’ rapid breakdown into smaller fragments to yield the largest possible surface area available for dissolution media [64]. The disintegration time of all LSTs (Y_2_) was in the range from 0.26 to 6.29 min for LS-4 and LS-9, respectively, as shown in Table 3.

Figure 5d revealed that there is an inverse relationship between the pattern of disintegration time (Y_2_) and superdisintegrant concentration (X_3_), i.e., when the X_3_ increases, the disintegration time decreases. As the X_3_ increases from 4 to 6% at the same level as the other factors, the disintegration time decreased from 4.02 to 0.57 min in LS-12 and LS-5, respectively, and from 3.86 to 0.78 min in LS-6 and LS-2, respectively. This trend can be confirmed by the decrease in the disintegration time from 3.46 to 0.26 min. in LS-10 and LS-4, respectively, as X_3_ increased. This finding is due to the rapid water-absorbing nature, as well as the capillary and swelling mechanisms, of polyplasdone that build up the pressure internally, leading to faster disintegration [65]. In addition, polyplasdone polymers are closely cross-linked homopolymers of polyvinyl pyrrolidones with a porous particle structure that allows them to quickly absorb liquids into the tablet by capillary action and to produce rapid volume enlargement and hydrostatic pressures that result in tablet disintegration. It was reported that polyplasdone has a non-ionic structure, which prevents its binding to ionic drug moieties. Moreover, polyplasdone does not form a gel at higher concentrations, and, for this reason, it is also used to enhance the solubility of drugs and improve their dissolution [66].

A similar finding was observed in the relationship between the disintegration time (Y_2_) and the liquid load factor (X_1_). Figure 5c reveals that increasing the liquid load factor (X_1_) in the LSTs showed an antagonistic effect on the tablet disintegration. The increase in X_1_ from 0.2 to 0.4 is always accompanied by a decrease in the disintegration time of tablets. At the same level of both X_2_ and X_3_ and increasing the X_1_ from 0.2 to 0.4, the disintegration time decreased from 2.54 to 1.13 min in LS-1 and LS-7, respectively, and from 1.04 to 0.26 min. in LS-3 and LS-4, respectively. This observation could be confirmed by the decrease in Y_2_ from 2.32 to 1.12 min for LS-8 and LS-11, respectively. Specifically, increasing the L_f_ of the LSTs increases the amount of liquid used and significantly increases the wetting characteristics and surface area of the drug, and increases the accessibility of the drug to be easily disintegrated from its LSTs, and this subsequently accelerates its disintegration [67].

It was evident that, when the percentage of X_3_ and X_1_ increases in the LSTs, the disintegration time of the prepared LSTs will significantly decrease with *p*-values of 0.0001 and 0.0016 for X_3_ and X_1_, respectively, as presented in the Pareto chart (Figure 4). The prediction Equation (8) of disintegration time value is:

Disintegration time (Y_2_) = 40.325 − 46.625 X_1_ + 0.302 X_2_ −10.901 X_3_ + 23.791 X_1_^2^ − 0.105 X_1_X _2_ + 5.125 X_1_X_3_ − 0.008 X_2_^2^ − 0.018 X_2_X_3_ + 0.768 X_3_^2^ (8)

The disintegration time of formulations containing 6% of X_3_, such as LS-2, LS-4, LS-3, and LS-5, had shorter disintegration times of 0.26, 0.57, 0.78 and 1.04 min, respectively, while the disintegration time of the formulations containing 4% of X_3_ disintegrated in 6 min.

#### 3.3.3. Effect of Independent Variables on the Dissolution Efficiency of TDL and DPX (Y_3_ and Y_4_)

**Figure 6:**
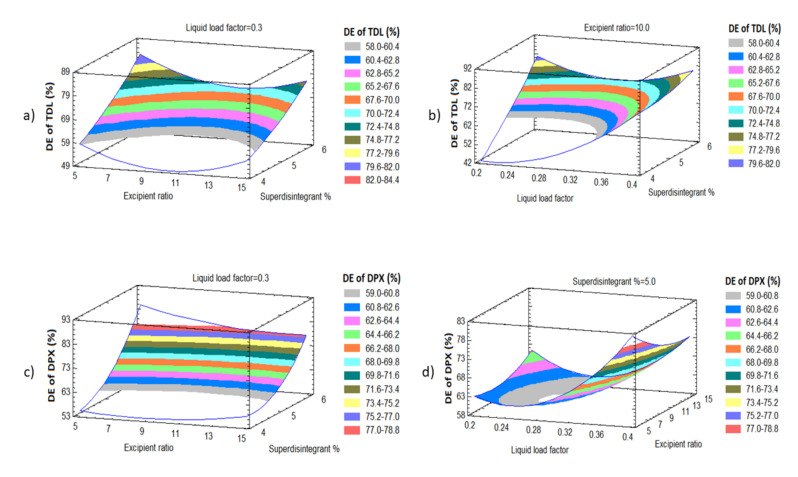
Response surface plots showing the effect of X_1_, X_2_ and X_3_ (**a**,**b**) on the dissolution efficiency of TDL and (**c**,**d**) on the dissolution efficiency of DPX.

Figure 6 revealed that the dissolution efficiencies of TDL and DPX (Y_3_ and Y_4_) were significantly affected by both (X_1_) and (X_3_). There is a synergistic relationship between and liquid load factor (X_1_) and superdisintegrant concentration (X_3_), i.e., when the X_1_ and X_3_ increase, the dissolution efficiency increases as well. As the X_1_ increases from 0.2 to 0.4 at the same level as the other factors, Y_3_ increases from 70.28 to 78.25% in LS-1 and LS-7, respectively, and from 74.85 to 78.68% in LS-3 and LS-4, respectively. This trend can be confirmed by the increase in Y_3_ from 70.91 to 82.18% in LS-8 and LS-10, respectively, as X_1_ increased from 0.2 to 0.4. When X_3_ increases from 5 to 15 at the same level as other factors, Y_3_ increases from 44.37 to 74.85% in LS-9 and LS-3, respectively. This trend can be confirmed by the increase in Y_3_ from 55.25 to 87.87% in LS-6 and LS-2, respectively. The same finding was observed for the dissolution efficiency of DPX (Y_4_). In addition, there is a significant synergistic effect of X_3_ and X_1_ on Y_4_, as presented in Figure 6c,d. These significant effects can be found in LS-6 and LS-2 when increased from 54.19 to 91.81%, respectively, by increasing X_1_ from 5 to 15 at the same level of other factors. Other instances can be confirmed by the increase in the dissolution efficiency of DPX from 51.42 to 82.02% in LS-9 and LS-3, respectively.

It was detected that both DE_60_ of TDL and DE_60_ of DPX are controlled by the percentage of liquid load factor X_1_, which had a significant synergistic effect on Y_3_ and Y_4_ with *p* values of 0.0233, and 0.0417, respectively (Table 4 and Figure 6c,d). In addition, the superdisintegrant concentration (X_3_) was found to have a significant synergistic effect on Y_3_ and Y_4_ with *p*-values of 0.0045 and 0.003, respectively. Model Equations (9) and (10) to predict both responses are given below:

DE_60_ of TDL (Y_3_) = 6.733 − 40.666 X_1_ − 5.043 X_2_ + 18.564 X_3_ + 890.687 X_1_^2^ − 1.647 X_1_X_2_ − 80.665 X_1_X_3_ + 0.309 X_2_^2^ − 0.213 X_2_ X _3_ + 1.897 X_3_^2^ (9)

DE_60_ of DPX (Y_4_) = 93.8799 − 86.635 X_1_ + 2.185 X_2_ − 28.633 X_3_ + 885.571 X_1_^2^ − 6.158 X_1_X_2_ − 63.605 X_1_X_3_ + 0.096 X_2_^2^ − 0.499 X_2_ X_3_ + 6.521 X_3_^2^ (10)

The dissolution profiles of TDL and DPX from the LS formulations indicated that the initial and cumulative TDL and DPX release increased markedly in the formulations with a high percentage of X_3_. An increase in X_3_ from 4 to 6%, at the same levels of other factors, led to an increase in Y_3_ from 44.37 to 87.871 for LS-9 and LS-2, respectively. The same observation was found in LS-9 and LS-3 by the increase in Y_3_ from 44.3 to 74.85, respectively. In addition, this finding could be confirmed by the increase in Y_3_ from 51.63 to 79.42% for LS-12 and LS-5, respectively. The same formulations exposed a similar behavior regarding the DE_60_ of DPX. This could be due to the release of surface-bound DPX from LSTs that explain the initial rapid release phase. Moreover, a direct relationship was observed between polyplasdone% and the DE_60_ of DPX. The DE_60_ decreased from 91.8 to 55.2% in LS-2 and LS-6, respectively, when the polyplasdone% decreased from 6 to 4% at the same level of X_1_ and X_2_. Additionally, Y_4_ decreased from 82 to 51% in LS-3 and LS-9, respectively, by decreasing X_3_ from 6 to 4% at the same level of X_1_ and X_2_. Moreover, this finding could be confirmed by the decrease in Y_3_ from 86.74 to 81.88% for LS-4 and LS-10, respectively, due to the decrease in X_3_ from 6 to 4% at the same level of X_1_ and X_2_. The molecularly dispersed drug in the solvent used in the prepared LSTs explains this dissolution behavior due to greater surface area in contact with the dissolution media that endorses the penetration of LSTs and improves the drug dissolution, which consequently increases the DE_60_ of both APIs.

### 3.4. Prediction of the Optimized Liquisolid Formulation

To compromise the investigated responses in an attempt to find the optimum combination of factors’ levels, multiple response optimization was performed. Consequently, the desirability function over the specified design space of the obtained data will be maximized. Table 5 demonstrates the optimal calculated independent variables. The optimal combination of these factors ensured the desired hardness, disintegration time, and dissolution efficiency for both TDL and DPX. Furthermore, it was previously recognized that the higher dissolution efficiency exhibited by LSTs may also designate the improved oral bioavailability due to an increase in the wettability and the surface area of APIs accessible for dissolution [68,69].

### 3.5. In-Vivo Pharmacokinetics Evaluation on Human Volunteers

**Figure 7:**
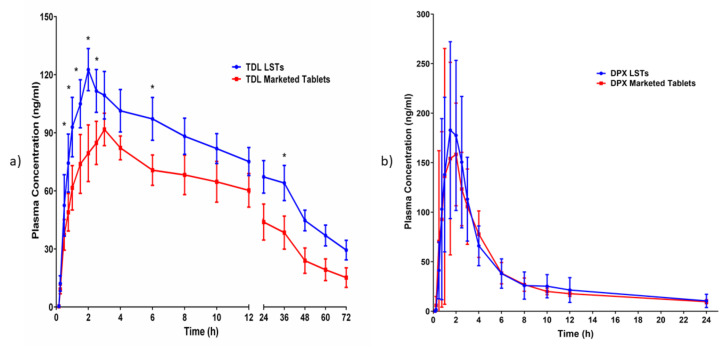
The mean plasma concentration–time profiles of (**a**) TDL after oral administration of a single oral dose (5 mg) of the marketed tablet and optimized liquisolid tablet, (**b**) DPX after oral administration of a single oral dose (30 mg) of the marketed tablet and optimized liquisolid tablet. * Significant difference at *p* < 0.05.

Figure 7 displayed the plasma concentration–time curve profiles after oral administration of optimized LSTs compared to the marketed tablets. All the involved volunteers have fully completed the clinical study. The pharmacokinetic parameters of the clinical study are depicted in Table 6.

The results indicated that the maximum plasma concentration (C_max_) of TDL in LSTs was 122.61 ng/mL within 2 h (T_max_), compared to the marketed tablets, which reach 91.72 ng/mL after 3 h. These findings indicated that the LST reached the maximum plasma concentration faster than the marketed tablet and consequently produced the rapid onset of therapeutic action. Amazingly, the optimized LST formulation achieved this C_max_ of the marketed tablet after only 1 h which revealed that LSTs formulation improved the rate and extent of TDL absorption compared to the marketed tablet. In addition, LSTs showed higher AUC in comparison to the marketed tablets. The improved absorption of LSTs was probably due to the enhanced solubilization of the drugs and the high surface area available for absorption. The dissolved drug in LSTs can be directly absorbed, with a short time for the dissolution step which is considered the rate-limiting step for drug absorption in BCS Class II compounds. Improvement of the dissolution rate can lead to a significant increase in oral absorption and subsequently enhance oral bioavailability [70,71]. The relative bioavailability of TDL of LSTs was larger (149.77%) than that of the marketed tablet. In addition, ANOVA of the data showed that there are significant differences (*p*-value < 0.05) among the samples taken at 0.75, 1, 1.5, 2, 2.5, 6 and 36 h from the two groups of volunteers, indicating the significant improvement achieved by the LSTs. The unpaired *t*-test with Welch’s correction discovered that there is a significant difference between the C_max_ and AUC_0–t_ of both groups with *p*-values of 0.0198 and 0.0280, respectively. Regarding DPX, despite the higher relative bioavailability of DPX of the LST (115.72%) than of the marketed tablet, an unpaired *t*-test with Welch’s correction revealed that the LS formulation did not differ significantly from the marketed tablets concerning C_max_, T_max_ and AUC at *p* < 0.05.

The optimized LS formulation containing 5 mg of TDL and 30 mg of DPX was compared with the marketed tablets at the same doses. The relative bioavailability of TDL was increased with rapid onset of action, as reflected by the shorter time to reach the T_max_. This result proves that the rapidity of onset of action, and the duration as well as the plasma drug concentration, are suitable for the treatment of male sexual dysfunction.

## 4. Conclusions

From the obtained results, we could conclude that BBD was successfully implemented in the optimization of the formulation factors to produce an optimized combined-dose of TDL and DPX LST with acceptable mechanical properties, short disintegration time, and good dissolution profile. The statistical analysis suggested the combination of the factor levels of 0.2 of the liquid load factor, 11.82 of the excipient ratio and 5.11% of polyplasdone XL-10 in the preparation of the optimized formulation. The pharmacokinetic evaluation revealed a significant improvement in the drug bioavailability after oral administration owing to the enhanced drug solubility and absorption. The maximum plasma concentration (C_max_) of TDL in LSTs was 122.61 ng/mL within 2 h (T_max_), compared to the marketed tablets which reach 91.72 ng/mL after 3 h. Amazingly, the optimized LST formulation achieved this C_max_ of the marketed tablet after only 1 h, which revealed that LSTs’ formulation improved the rate and extent of TDL absorption. In addition, the optimized formulation showed a significant difference between the C_max_ and AUC_0–t_ of both groups with *p*-values of 0.0198 and 0.0280, respectively. The improved absorption of LSTs was probably due to the enhanced solubilization of the drugs and the high surface area available for absorption. The dissolved APIs in the mixture of solvents used can be directly absorbed, with a short time for the dissolution step, which is considered the rate-limiting step for drug absorption in BCS Class II compounds. Improvement of the dissolution rate leads to a significant increase in oral absorption and a subsequent enhancement of oral bioavailability. The relative bioavailability of TDL and DPX of LSTs was larger (170.6% and 117.05%) than that of the marketed tablet. Finally, these findings reveal that the onset was rapid enough, and the duration and the concentration achieved a level suitable to overcome male sexual dysfunction. So, the developed combined-dose LS formulation could be a promising approach in the treatment of male sexual dysfunction, particularly for diabetic patients.

## Figures and Tables

**Table 1:** Composition of TDL and DPX Liquisolid formulations based on the Box–Behnken design.

Formula Code	Avicel, Q	Silica, q	Liquid Medication, W		TDL	DPX	Methocel 3%	Magnesium Trisilicate 5%	Polyplasdone XL-10
			PEG 200	Labrasol					
	(g)								
LS-1	5	0.3	0.795	0.795	0.1	0.6	0.22	0.379	0.455
LS-2	5	0.3	1.06	1.06	0.1	0.6	0.24	0.406	0.406
LS-3	5	0.3	0.53	0.53	0.1	0.6	0.24	0.353	0.353
LS-4	5	0.5	0.55	0.55	0.1	0.6	0.21	0.365	0.438
LS-5	5	1.0	1.2	1.2	0.1	0.6	0.27	0.455	0.455
LS-6	5	0.5	1.1	1.1	0.1	0.6	0.27	0.420	0.504
LS-7	5	0.5	1.1	1.1	0.1	0.6	0.25	0.420	0.336
LS-8	5	1.0	0.9	0.9	0.1	0.6	0.25	0.425	0.510
LS-9	5	1.0	0.9	0.9	0.1	0.6	0.25	0.425	0.340
LS-10	5	1.0	0.795	0.795	0.1	0.6	0.24	0.414	0.331
LS-11	5	1.0	0.55	0.55	0.1	0.6	0.23	0.390	0.312
LS-12	5	1.0	0.6	0.6	0.1	0.6	0.23	0.395	0.395
LS-13	5	0.5	0.825	0.825	0.1	0.6	0.23	0.392	0.392
LS-14	5	0.5	0.825	0.825	0.1	0.6	0.23	0.392	0.392
LS-15	5	0.5	0.825	0.825	0.1	0.6	0.23	0.392	0.392

**Notes:** L_f_ is the liquid load factor which is calculated as L_f_ = W/Q. R is the excipient ratio which is calculated as R = Q/q. Where: W is the weight of liquid medication, Q is the amount of carrier material and q is the amount of coating material. TDL is tadalafil and DPX is dapoxetine.

**Table 2:** Pre-compression and post-compression properties of liquisolid formulations.

**Formula Code**	**Pre-Compression Properties**				**Post-Compression Properties**					
	**Hausner Ratio**	**Carr’s Index**	**Angle of Repose**	**Type of Flow**	**Friability (%)**	**Hardness (*n*)**	**Weight (mg)**	**Disintegration Time (min)**	**Content of TDL%**	**Content of DPX%**
LS-1	1.1	14.5	29	Excellent	0.461	58.02	200	2.54	95.2	95.8
LS-2	1.2	19.7	26	Good	0.151	38.91	227	0.78	95.9	97.6
LS-3	1.09	8.78	21	Excellent	0.357	56.44	199	1.04	99.6	99.8
LS-4	1.1	5.1	26	Excellent	0.356	35.28	207	0.26	101.1	99.9
LS-5	1.1	10	30	Excellent	0.334	47.14	259	0.57	97.3	98.2
LS-6	1.2	12	35	Good	0.300	41.85	239	3.86	97.8	100.1
LS-7	1.18	15.4	30	Excellent	0.294	36.75	235	1.13	102.2	99.3
LS-8	1.19	16	34	Fair	0.253	48.51	244	2.32	98.1	100.1
LS-9	1.21	17.5	36	Fair	0.245	49.69	238	6.29	99.0	97.3
LS-10	1.15	13.4	31	Good	0.268	33.81	234	3.46	103.2	95.6
LS-11	1.31	25	41	Passable	0.219	32.14	220	1.12	95.6	95.5
LS-12	1.2	20	34	Fair	0.193	45.96	226	4.02	98.8	98.6
LS-13	1.17	14	34	Good	0.170	44.2	223	1.86	101.3	102.2
LS-14	1.14	12.5	31	Good	0.164	43.81	237	1.62	102.1	99.9
LS-15	1.3	25	41	Passable	0.086	42.63	222	1.79	100.1	99.6

**Table 3:** Box–Behnken design matrix of the liquisolid formulations showing the independent and dependent variables.

Formula Code	Independent Variables			Dependent Variables			
	X_1_	X_2_	X_3_	Y_1_	Y_2_	Y_3_	Y_4_
LS-1	0.2	15.0	5.0	58.02	2.54	70.285	71.858
LS-2	0.3	5.0	6.0	38.91	0.78	87.871	91.812
LS-3	0.2	10.0	6.0	56.44	1.04	74.851	82.016
LS-4	0.4	10.0	6.0	35.28	0.26	78.682	86.741
LS-5	0.3	15.0	6.0	47.14	0.57	79.418	78.323
LS-6	0.3	5.0	4.0	41.85	3.86	55.255	54.192
LS-7	0.4	15.0	5.0	36.75	1.13	78.256	74.047
LS-8	0.2	5.0	5.0	48.51	2.32	70.918	62.051
LS-9	0.2	10.0	4.0	49.69	6.29	44.375	51.427
LS-10	0.4	10.0	4.0	33.81	3.46	80.555	81.886
LS-11	0.4	5.0	5.0	32.14	1.12	82.183	76.889
LS-12	0.3	15.0	4.0	45.96	4.02	51.630	51.115
LS-13	0.3	10.0	5.0	44.20	1.86	57.564	61.947
LS-14	0.3	10.0	5.0	43.81	1.62	56.571	57.015
LS-15	0.3	10.0	5.0	42.63	1.79	62.234	61.171

**Table 4:** Statistical analysis of variance (ANOVA) of the responses (Y_1_–Y_4_) results.

Factors	Hardness (Y_1_)		Disintegration Time (Y_2_), min		Dissolution Efficiency for TDL (Y_3_), %		Dissolution Efficiency for DPX (Y_4_), %	
	Estimate	*p*-Value	Estimate	*p*-Value	Estimate	*p*-Value	Estimate	*p*-Value
X_1_	−18.671	0.0001 *	−1.555	0.0016 *	14.791	0.0233 *	13.020	0.0417 *
X_2_	6.615	0.0026 *	0.045	0.8643	−4.302	0.3913	−2.420	0.6344
X_3_	1.615	0.2337	−3.745	0.0001 *	22.415	0.0045 *	25.009	0.0034 *
X_1_^2^	0.648	0.7271	0.476	0.2529	17.814	0.0460 *	17.711	0.0535
X_1_ X_2_	−2.451	0.2061	−0.105	0.7786	−1.647	0.8096	−6.158	0.4044
X_1_ X _3_	−2.641	0.1783	1.025	0.0339 *	−16.133	0.0553	−12.721	0.1188
X_2_^2^	−0.031	0.9863	−0.434	0.2915	15.427	0.0710	4.788	0.5267
X_2_ X_3_	2.061	0.2764	−0.185	0.6234	−2.128	0.7560	−4.991	0.4939
X_3_^2^	−0.131	0.9431	1.536	0.0087 *	3.794	0.5983	13.042	0.1232
R^2^	98.269		98.318		91.366		90.764	
Adj. R^2^	95.152		95.290		75.825		74.139	

Note: * Significant effect of factors on individual responses. Abbreviations**:** X_1_, liquid load factor; X_2_, excipient ratio; X_3_, superdisintegrant concentration; X_1_ X _2_, X_1_X_3_, X_2_ X_3_, the interaction term between the factors; X_1_^2^, X_2_^2^ and X_3_^2^ are the quadratic terms of the factors; R^2^, R-squared; and Adj-R^2^, Adjusted R-squared.

**Table 5:** Optimal calculated independent variables and observed, predicted, and residual values for dependent variables.

Independent Variables	Optimum	Dependent Variables	Predicted Values	Observed Values	Residuals
Liquid load factor	0.2	Hardness	55.2	54.1	1.1
Powder excipient ratio	11.82	Disintegration time	2.7	2.8	0.1
Superdisintegrant concentration	5.11	Dissolution efficiency for TDL	66.4	68.6	−2.2
		Dissolution efficiency for DPX	74.5	77.2	−2.7

**Table 6:** Pharmacokinetic parameters of TDL and DPX in the optimized Liquisolid tablet compared to TDL and DPX in the marketed tablets after oral administration of a single dose to human volunteers (mean ± SD; *n*  =  6).

PK Parameter	Unit	TDL		DPX	
		LSTs	Marketed Tablets	LSTs	Marketed Tablets
Lambda_z	1/h	0.104 ± 0.096	0.051 ± 0.034	0.065 ± 0.010	0.051 ± 0.006
t_1/2_	h	18.523 ± 22.737	17.544 ± 8.557	10.951 ± 1.879	11.683 ± 1.989
Tmax	h	2 ± 0	3.0 ± 0	1.667 ± 0.289	1.833 ± 0.289
Cmax	ng/ml	122.612 * ± 10.876	91.719 ± 8.347	186.154 ± 83.741	171.063 ± 71.830
AUC_0–t_	ng/mL∙h	4484.953 * ± 408.147	2994.611 ± 591.332	919.633 ± 397.978	794.699 ± 195.442
AUC_0–inf_	ng/mL∙h	5231.316 ± 1579.022	3066.42 ± 573.078	1096.416 ± 521.708	936.702 ± 170.519
AUMC_0–inf_	ng/mL∙h^2^	241,586.7 ± 175,386.7	97,771.74 ± 20,029.83	13,201.782 ± 9197.529	10,844.661 ± 983.277
MRT_0–inf_	h	42.650 ± 17.812	31.878 ± 2.155	11.492 ± 3.034	11.915 ± 2.817
Vz/F	(mg)/(ng/mL)	0.021 ± 0.022	0.042 ± 0.023	0.504 ± 0.251	0.562 ± 0.185
Cl/F	(mg)/(ng/mL)/h	0.001 ± 0.0002	0.002 ± 0.0003	0.033 ± 0.018	0.033 ± 0.006

* Significant difference at *p* < 0.05 (unpaired *t*-test with Welch’s correction). PK, pharmacokinetic; TDL, tadalafil; DPX, dapoxetine; AUC, area under the time–concentration curve; Cmax, maximum plasma concentration; Tmax, the time required to reach the Cmax; Lambda_z, elimination rate constant; MRT, mean residence time; Vz/F, Apparent volume of distribution during terminal phase; Cl/F, apparent total clearance of the drug from plasma after drug oral administration.
